# Unusual accelerated rate of deletions and insertions in toxin genes in the venom glands of the pygmy copperhead (*Austrelaps labialis*) from kangaroo island

**DOI:** 10.1186/1471-2148-8-70

**Published:** 2008-02-28

**Authors:** Robin Doley, Nguyen Ngoc Bao Tram, Md Abu Reza, R Manjunatha Kini

**Affiliations:** 1 Department of Biological Sciences, Faculty of Science, National University of Singapore, Singapore 117543, Singapore; 2 Protein Science Laboratory, Department of Biological Sciences, National University of Singapore, 14 Science Drive 4, Block S3, # 03-17, 117543, Singapore

## Abstract

**Background:**

Toxin profiling helps in cataloguing the toxin present in the venom as well as in searching for novel toxins. The former helps in understanding potential pharmacological profile of the venom and evolution of toxins, while the latter contributes to understanding of novel mechanisms of toxicity and provide new research tools or prototypes of therapeutic agents.

**Results:**

The pygmy copperhead (*Austrelaps labialis*) is one of the less studied species. In this present study, an attempt has been made to describe the toxin profile of *A. labialis *from Kangaroo Island using the cDNA library of its venom glands. We sequenced 658 clones which represent the common families of toxin genes present in snake venom. They include (a) putative long-chain and short-chain neurotoxins, (b) phospholipase A_2_, (c) Kunitz-type protease inhibitor, (d) CRISPs, (e) C-type lectins and (f) Metalloproteases. In addition, we have also identified a novel protein with two Kunitz-type domains in tandem similar to bikunin.

**Conclusion:**

Interestingly, the cDNA library reveals that most of the toxin families (17 out of 43 toxin genes; ~40%) have truncated transcripts due to insertion or deletion of nucleotides. These truncated products might not be functionally active proteins. However, cellular trancripts from the same venom glands are not affected. This unusual higher rate of deletion and insertion of nucleotide in toxin genes may be responsible for the lower toxicity of *A. labialis *venom of Kangroo Island and have significant effect on evolution of toxin genes.

## Background

Australian elapids are among the most venomous land snakes of the world. Various bioactive peptide have been purified and characterized from these venoms and a comprehensive survey of the bioactive proteins present in a number of these snake venoms have been reported recently [1]. Snakes in *Austrelaps *genus are widely distributed in Australia, but are moderately toxic compared to other elapids [2]. Presently three species of *Austrelaps *(commonly referred as copperheads) are known – Lowland copperhead (*Austrelaps superbus*), Highland copperhead (*Austrelaps ramsayi*) and Pygmy copperhead (*Austrelaps labialis*). So far, only a small number of proteins have been isolated and characterized from *A. superbus *venom. They are mostly phospholipase A_2 _(PLA_2_) enzymes [3-5] and cobra venom factor-like protein [6]. However, no significant data on the venoms of other two species are available. Pigmy copperhead (*A. labialis*) is smaller in size as compared to *A. superbus *and *A. ramsayi *[7] and it has distinguishable white bars to its upper lips, circular eyes and yellowish-brown iris [8]. They mainly feed on small lizards and frogs [7] and the LD_50 _of their venom is 1.3 mg/kg [8]. In the present study, we have attempted to profile venom components of *A. labialis *to define its composition and to look for novel proteins.

Profiling venom toxins can be achieved via transcriptomics or proteomics approach. In former approach, the transcripts are directly sequenced from a cDNA library constructed from the venom gland, whereas in the latter approach venom proteins are separated using various techniques like LCMS, 2D gel electrophoresis and HPLC. We have obtained toxin profile of pygmy copperhead venom by constructing of cDNA. The data revealed that neurotoxins and PLA_2 _are the most abundant proteins in this venom. Interestingly, most of the toxin families in this cDNA library have truncated transcripts. We propose that the lower venom toxicity and subsequent decreased size of these snakes could be due to unusually high degree of deletions or insertions (~40%) in their toxin genes resulting in truncated, most likely inactive products.

## Methods

### Construction of library and DNA sequencing

Venom glands were dissected and extracted from an euthanized *A. labialis *snake captured in Kangaroo Island, South Australia (Venom supplies Pte Ltd, Tanunda, South Australia). Total RNA was extracted using RNeasy^® ^mini kit from Qiagen (Valencia, CA, USA). The purity and the concentration were spectrophotometrically determined. First strand cDNAs were synthesized from 150 ng of total RNA according to protocol of Creator™ SMART™ cDNA library construction kit obtained from Clontech Laboratories (Palo Alto, CA, USA). Amplification of full-length double-stranded cDNA was carried out using PCR-based protocol. Double-stranded cDNA PCR products (100 bp-10 kb) were purified and subjected to TA cloning. Ligation products were transformed into the competent TOP10 *E. coli *strain obtained from Invitrogen (Carlsbad, CA, USA,) and plated on LB/Amp/IPTG/X-gal for blue/white screening. A bidirectional pGEM^®^-T Easy vector system obtained from Promega (Madison, WI, USA) plasmid-based cDNA library was titered with 0.8 × 10^6 ^CFU/ml. Individual colonies were picked randomly and the presence of insert was confirmed by EcoRI digestion. Only clones containing inserts larger than 200 bp in length were selected for further DNA sequencing.

DNA sequencing reactions were carried out using the ABI PRISM^® ^BigDye^® ^terminator cycle sequencing ready reaction kit (BDV3.1) according to manufacturer's instructions (Applied Biosystem, Foster City, CA, USA). DNA sequencing was carried out using ABI PRISM^® ^3100 automated DNA sequencer. Sequencing was repeated at least twice in all singletons to make sure there was no error created by sequencing step.

### Cloning snake venom protein with two Kunitz domains

PCR amplification of protein containing two Kunitz-type domain from RNA pool was performed using a forward primer designed from the signal peptide-encoding region (5'-CGATGACGCGCGAGAAAAG-3') and the reverse primer from library construction kit (5'-ATTCTAGAGGCCGAGGCGGCCGACATG-d(T)30-3'). Long PCR enzyme mix (Fermentas, Burlington, Ont., Canada) was used in the amplification reaction. PCR was performed as follows: 35 cycles of one step each at 95°C for 15 s, 58°C for 15 s, 68°C for 3 min followed by a final extension step at 68°C for 10 min. PCR products were subjected to 1% agarose gel and visualized using ethidium bromide staining. Amplified PCR products were gel purified and cloned to pGEM^®^-T Easy vector system and sequenced subsequently.

### Analysis of sequence data by bioinformatics tools

The DNA sequences were analyzed after trimming the adaptor sequences (GENE RUNNER software) and the putative functions of the gene products were predicted by batch BLASTing sequence results in GRID blast system. The signal peptide was predicted using online SignalP 3.0 server. Clustering of sequences was performed using FastGroupII [9] and sequences were aligned using ClustalW [10]. Jalview [11] was employed to make some necessary editing on sequence alignments. Phylogenetic trees were generated using PHYML [12], which uses maximum likelihood method to build phylogenetic tree.

## Results and discussion

### Compositions of cDNA library

**Figure 1:**
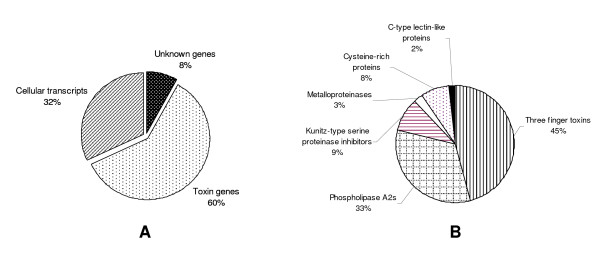
**Composition of a cDNA library from *A. labialis *venom gland**. A) Relative abundance of genes sequenced from the cDNA library; B) Relative abundance of the toxin genes in the cDNA library.

We randomly selected and isolated 780 clones. Of these only 658 clones, which had larger than 200 bp inserts, were sequenced and analyzed. These ESTs were categorized into different clusters on the basis of sequence similarities. Members of each clusters has been sequenced completely and the full length sequences were submitted to NCBI with accession numbers from EU003085 to EU003110, EU012449 and EU012450. BLAST search against nucleotide, protein and EST database, revealed that 60% of the EST belong to putative toxins, while 32% belong to cellular transcripts and 8% of the EST were unknown transcripts (Fig. 1A). These relative ratios may change, if more EST clones are sequenced and analyzed.

### Toxin genes

Putative toxin genes found in the cDNA library were further grouped based on their sequence similarities to known toxin superfamilies (Fig. 1B). In total, we found six superfamilies of the toxin genes in the cDNA library of *A. labialis*. These families, in order of abundance, are three-finger toxin family (193 clones), phospholipase A_2 _family (139 clones), Kunitz-type proteinase inhibitor family (38 clones), cysteine-rich protein family (32 clones), metalloproteinase family (12 clones) and C-type lectin family (7 clones) (Fig. 1B).

### Three-finger toxin family

**Figure 2:**
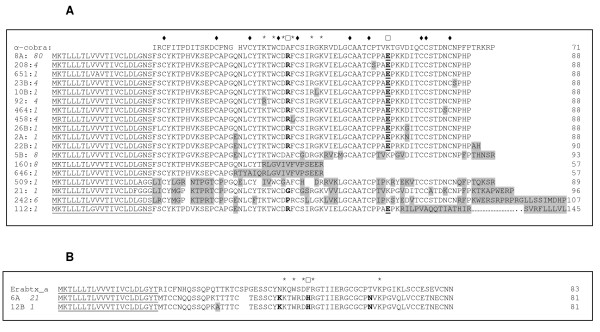
**Three-finger toxins in *A. labialis *venom gland**. **A) **Alignment of long-chain neurotoxins with α-cobratoxin (α-cobra) [64]. The changed in amino acids are highlighted in grey which is either due to addition or deletion of nucleotide. ◆, conserved Cysteine residues; *, residues involved in binding to nicotinic acetylcholine receptor (nAChR); and □, residues Ala28 and Lys49 involved in binding to α7 receptor and nAChR receptor, respectively. Residues that are different from consensus sequence of *A. lablialis *toxins are highlighted. The truncated transcripts and the elongated protein product (clone 112; for brevity, functionally unimportant parts of the sequence are not shown and the dots indicate the missing segments). The number of clones is shown in italics and the predicted signal peptide using SignalP 3.0 is underlined (The signal peptide for α-cobrotoxin is not available). **B) **Alignment of two cDNA-deduced peptide representatives of one cluster and one singleton of putative short-chain neurotoxin with Erabutoxin a (Erabtx_a) [65]. The gaps in clone 6A and 12B are represented by blank spaces. *, residues involved in binding to nicotinic acetylcholine receptor (nAChR). The substitution of Thr to Ala in clone 12B is highlighted. Phe32 (□) of erabutoxin a involved in binding to nAChR receptor which is substituted with His32 in both the clones of *A. labialis *is highlighted. The number of clones is shown in italics and the predicted signal peptide using SignalP 3.0 is underlined.

Three-finger toxin family is a well characterized non-enzymatic polypeptide family named after their canonical protein folds. Members of this family contain about 60–74 amino acid residues [13-16] and exhibit various pharmacological activities such as neurotoxic, cytotoxic, cardiotoxic, anticoagulant and antiplatelet effects [13-15]. Three-finger family forms the major group of toxins (45% abundance) in this cDNA library, and majority of mature proteins are 66–69 amino acid residues in length. We identified a total of 166 sequences (8 clusters and 10 singletons) with 60%–80% identities to known long-chain neurotoxins (Figure 2A) and 27 sequences (1 cluster and 1 singleton) with 60%–85% identical to short-chain neurotoxins (Figure 2B).

#### Long-chain neurotoxins

Clone 8A (80 clones) is the most dominant long-chain neurotoxin and other clones (208, 23B, 10B, 92, 464, 458 and 26B) are its isoforms with few amino acid substitutions (Figure 2A). Most of these substitutions are due to single nucleotide changes. Since these isoforms are represented by many clones, these sequence differences are not artifacts of sequencing. However, clone 5B has 10 substitutions and hence may have distinct specificity compared to other long-chain neurotoxins. Further, a dinucleotide (GT) deletion in front of the typical stop codon position followed by a dinucleotide (CT) insertion (Additional file 1) in the gene resulted in a frame shift and additional five amino acid residues in clone 5B (Figure 2A). However, two clones (160 and 646) are distinctively shorter due to deletion of nucleotides A131 and G111 respectively (see Additional file 1), resulting in the frame shift and truncated peptides.

There were eight clones identical to clone 160 suggesting that this observation is not due to sequencing error. The segments after the deletion show significant identity with other complete transcripts. Similar phenomenon was observed in sea snake *Aipysurus eydouxii *in which a dinucleotide deletion leads to the truncation of the only three-finger neurotoxin found in the venom gland library and the loss of toxicity [17]. In contrast, in clone 112 deletion of A224 leads to a potentially much longer protein product. In this case, three of the conserved cysteine residues at the C-terminal end are lost due to the frame shift. Three distinct neurotoxin sequences (clones 21, 242 and 509) compared to clone 8A and its closer sibling toxins were also found in the cDNA library. Clones 21 and 242 encode for slightly longer proteins with 75 and 86 amino acid residues respectively as compared to other clones. In these cases there is a single nucleotide substitution at consensus stop codon (TAA) present in the majority of these putative long-chain neurotoxins. In clone 509, a dinucleotide (TA) deletion in the stop codon followed by a dinucleotide (CA) insertion and a dinucleotide deletion (AC) has been found.

Most of the putative full length long-chain neurotoxins possess the functionally conserved residues such as Lys23, Trp25, Asp27, Phe29, Arg33 and Lys35 that are shown to participate in the binding to *Torpedo *nicotinic acetylcholine receptor (nAChR) in the case of α-cobratoxin from *Naja naja *venom [18]. However, Lys49 (corresponding to position 50 in *A. labialis *toxins) is replaced by the negatively charged Glu50 (Figure 2A). This charge reversal may significantly reduce its interaction with *Torpedo *nAChR as mutation of K49 to E49 in α-cobratoxin cause 53 fold decrease in binding affinity [18]. Further, neutrally charged Ala28 (corresponding to position 29 in *A. labialis *toxins) which is specific for binding to α7-nAChR in α-cobratoxin, is replaced by the positively charged Arg29 with the exception of clone 5B (Figure 2A). This substitution did not affect the binding affinity to the receptor in the case of α-cobratoxin [19].

**Figure 3:**
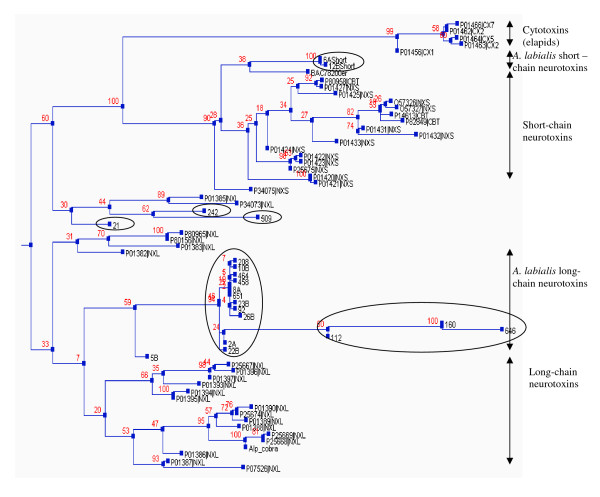
**Phylogenetic tree of elapid three-finger toxins**. Full length *A. labialis *long-chain and short-chain neurotoxins are encircled. Phylogram was generated by PHYML using maximum likelihood method.

The majority of *A. labialis *long-chain neurotoxins form a distinctive group almost completely separated from the other long-chain neurotoxins (Figure 3). Phylogenetically, this separation seems to start quite early in the evolution time frame as indicated by early branching pattern originating from the root. Interestingly, three distinct clones (21, 242 and 509) form a separate cluster as compared to other long-chain neurotoxins and appear to have a completely different phylogenetic origin.

#### Short-chain neurotoxins

Two representatives of short-chain neurotoxins, Clone 6A and 12B were found in the library (1 cluster with 21 clones and 1 singleton respectively). Their amino acid sequences are identical except for the substitution of Thr13 by Ala. Both short-chain neurotoxins have one additional Cys residue at the fourth position (Figure 2B). Appearance of Cys at 4^th ^position is observed for short-chain neurotoxins from sea snake venom [20]. They have functionally invariant residues such as Lys27, Trp29, Asp31, Arg33 and Lys47 which are important in the binding of erabutoxin a (*Laticauda semifasciata*) to *Torpedo *nAChR [21,22]. Interestingly, one of the binding residues, Phe32 found in erabutoxin-a, is replaced by His in *A. labialis *short-chain neurotoxins which might effect its binding to the receptor.

### Phospholipase A_2 _family

Phospholipase A_2 _(PLA_2_; EC 3.1.1.4) enzymes are esterolytic enzymes which hydrolyze glycerophospholipids at the *sn*-2 position of the glycerol backbone releasing lysophospholipids and free fatty acids. Snake venom PLA_2 _enzymes are one of the well studied superfamily of snake venom enzymes [23] which play an important role in immobilization and capture of prey. In addition to their role in digestion of preys, they exhibit variety of pharmacological effects such as neurotoxic, myotoxic, cardiotoxic, anticoagulant, antiplatelet and edema-inducing effects [24]. A total of 139 PLA_2 _cDNA clones were obtained which makes up the second abundant family. The deduced amino acid sequences are 65% to 97% identical to other PLA_2 _sequences and were grouped into three clusters and four singletons. All PLA_2 _cDNA sequences obtained from this snake belong to group IA.

Clones 243, 330, 563 and 488 are the most abundant clones in this library and are closely related to the PLA_2 _sequences of *A. superbus *[5] of the same genus. However, they are different from the other PLA_2 _enzymes due to the presence of three tryptophan residues in the β-wing. At present, role of these Trp residues are not known. These PLA_2 _enzymes together with five PLA_2 _isoforms of *A. superbus *(AAD56550, AAD56551, AAD56552, AAD56553, and AAD56554) [5] form a distinguishable cluster shown in phylogram generated by PHYML method (data not shown). This cluster is well separated from the other PLA_2 _enzymes having one tryptophan residue in the β-wing.

**Figure 4:**
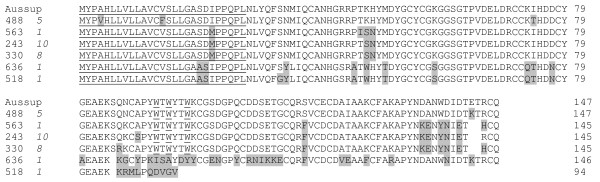
**Phospholipase A_2 _enzymes in *A. labialis *venom gland**. Alignment of PLA_2 _enzymes from *A. labialis *with *A. superbus *(Aussup) [5] is shown. Residues that are different from *A. superbus *PLA_2 _are highlighted. The three Trp (W) residues present in the β-wing of the *A. labialis *PLA_2 _enzyme are underlined. Clone 518 which encodes a truncated protein is also shown. The number of clones is shown in italics and the predicted signal peptide using SignalP 3.0 is underlined.

Clones 518 and 636 have significant difference in amino acid sequences of other *A. labialis *PLA_2 _enzymes. Clone 636 encodes for the full length protein sequence, whereas clone 518 encodes for a truncated product due to a single nucleotide (A) addition at position 247 (Additional file 2) (from ATG) leading to frame shift and premature truncation (Figure 4). These clones form a distinct cluster along with AAD56557 (PLA_2 _from *A. superbus*) (data not shown).

### Kunitz-type serine protease inhibitor family

Kunitz-type of protease inhibitors are 60 residues long which possess the conserved sequence motif of 6 cysteines (C [8X]C [15X]C [4X]YGGC [12X]C [3X]C) [25] and functionally belong to bovine pancreatic trypsin inhibitor (BPTI) family [26]. This family of snake venom protein is known to involve in coagulation, fibrinolysis and inflammation through interaction with various proteases [27].

**Figure 5:**
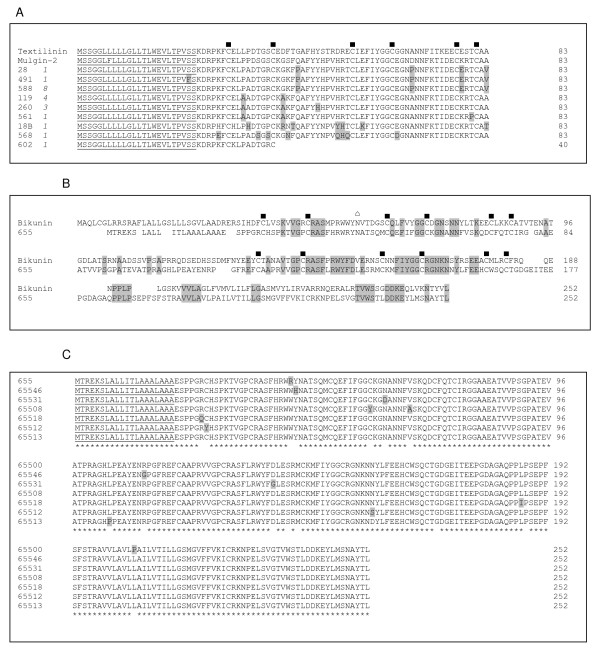
**Serine protease inhibitors in *A. labialis *venom gland**. **A) **Alignment of Kunitz-type serine protease inhibitors. ■, conserved six cysteine residues in kunitz domain. Residues that are different from the consensus sequence of *A. labialis *proteins are highlighted. The number of clones is shown in italics and the predicted signal peptide using SignalP 3.0 is underlined. **B) **Alignment of clone 655 (serine protease inhibitor containing two Kunitz domains) with bikunin [29]. Signal peptide of clone 655 is indicated by a vertical bar ( | ), ■, conserved six cysteine residues; , N-glycosylation site. Conserved residues are highlighted. **C) **Alignment of isoforms of two Kunitz-type domains. The presence of two Kunitz-type domains was further confirmed by PCR using gene specific primer and reverse primer from cDNA construction kit (for details, see Materials and methods). *, consensus residues and residues that are different from the consensus sequence are highlighted. The number of clones is shown in italics and the predicted signal peptide using SignalP 3.0 is underlined.

38 cDNA clones in this library belong to Kunitz-type serine protease inhibitor family. The sequences of this group are 77%–90% identical to mulgin isoforms (*Pseudechis australis*) and textilinin isoforms (*Pseudonaja textilis textilis*) (Figure 5A). Textilinins are shown to be distinct plasmin inhibitors and have a 60% bleeding reduction in murine model [28]. It will be interesting to study the role of these Kunitz-type serine proteases inhibitors in blood clotting cascade. However one of the clones, clone 602 was found to be truncated prematurely due to transversion of one nucleotide (A→T) at position 122 (from AAA encoding for Lys to TAA stop codon). This clone was resequenced to confirm the transversion is not due to sequencing error.

A unique clone (655) encoding protein with two tandem Kunitz-type protease inhibitor domains was found in this cDNA library. The entire cDNA sequence is 2,031 bp long (Figure 5B) and the deduced peptide has 252 amino acid residues with potential glycosylation site. Its presence was further confirmed by PCR using gene specific and reverse primer from cDNA construction kit. We sequenced 48 clones and seven isoforms with minor changes in the amino acid sequence were found (Figure 5C). They are 40% identical to bikunin, a serine proteinase inhibitor with two Kunitz domains [29]. Bikunin from human placenta exhibits a potent inhibitory effect to some serine proteases crucial in the blood coagulation and fibrinolysis, such as factor XIa and plasmin [30]. This is the first report of this novel member of Kunitz-type serine protease inhibitor family from snake venom.

### Cysteine-rich protein family

Cysteine-rich secretory proteins (CRISPs) are abundantly found in mammalian reproductive tracts and play important role in sperm maturation and immune system [31]. They have also been found in venoms of reptiles. The conserved sequence in CRISPs span through out the protein with 16 cysteine residues and 10 of them resides in the C-terminal end [32]. CRISPs have been purified and characterized from various snake venoms [32,32-34]. Functions of majority of these proteins are unknown. Some of them are known to block cyclic nucleotide-gated ion channels [35,36] while several others block potassium-stimulated smooth muscle contraction [33].

**Figure 6:**
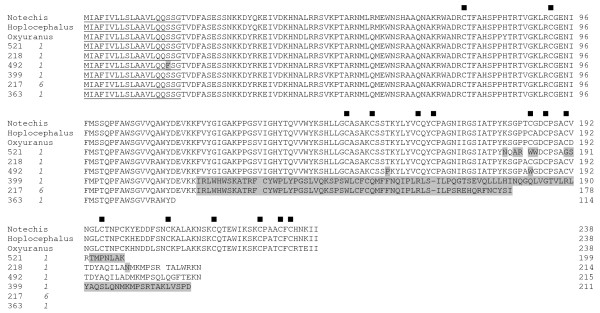
Alignment of CRISPs from *A. labialis Notechis scutatus*, *Hoplocephalus stephensii*, *Oxyuranus microlepidotus *[66]. ■, conserved cysteine. Residues that are different from the majority of sequences are highlighted. The number of clones is shown in italics and the predicted signal peptide using SignalP 3.0 is underlined.

32 cDNA clones encoding CRISPs making up 8% of the library has been observed. This family has one cluster and four singletons (Figure 6). Clone 521 has a dinucleotide deletion (CT) in position 549 and series of single nucleotide deletion of A in positions 537, 579, 595 and 597, single nucleotide deletion of T, C and G at position 575, 606 and 623 respectively. Clone 218 has two single nucleotide deletions of A579 and A648. Clone 492 has also two single nucleotide deletions of A579 and G623. Clones 217 and 399 have an insertion of one A at position 349 while clone 399 has another insertion of T in position 483. In case of clone 363 there is an insertion of T and A at position 341 and 349, deletions of T572, A579, A605, A610 and A621 (Additional file 3). Due to these insertions in clone 217 and 399 C-terminal end has encoded different amino acid residues as compared to other CRISPs. Clones 521, 218, 492 and 363 have several deletions in the ORF leading to frame shift and premature truncation. Thus, all of them are prematurely terminated losing the C-terminal domain where most of the conserved cysteine residues are found (Figure 6). Thus the truncated transcript might not be functional. However, the N-terminal region have high identity to snake venom CRISPs (53%–94%), but low identity to human, murine, frog CRISPs (less than 40%).

### Metalloproteinase family

Snake venom metalloproteinases are more abundantly found in viper venoms, but they are also reported from elapid family [37,38]. They are synthesized as zymogens in the venom gland and contain a propeptide which is cleaved off during maturation. They have a common zinc binding site with a consensus sequence of HEXXHXXGXXH [39]. They are classified into different types (P-I to P-IV) on the basis of the other domains that are present in these complexes [40]. This family of enzymes are responsible for haemorrhagic [41,42], local myonecrotic [43], antiplatelet [44], edema-inducing and other inflammatory effects [45,46]. We have found 12 clones which makes 3% of the entire cDNA library, however they code for only the signal peptide and the N-terminal region similar to trigramin precursor and mocarhagin (data not shown). This is due to the loss of two nucleotides at position 316 downstream of ATG leading to premature termination of the protein. Thus, if proteins of this family are present in the venom, they would have only partial propeptide without any other functional domains of metalloproteinase or disintegrin.

### C-type lectin family

C-type lectins are non-enzymatic proteins that bind to mono- and oligosaccharides in presence of Ca^2+ ^[47]. Generally they contain the highly conserved domain called the carbohydrate recognition domain (CRD) [48]. C-type lectins and related proteins have been frequently reported from Viperidae snake venoms. Venom C-type lectin related proteins are known to disrupt the normal functioning of haemostatic mechanism by interfering in the normal platelet receptor-ligand interaction, binding to coagulation factors and other important proteins in coagulation cascade [48]. Only few of them have been reported from elapids [49-53] including Australian elapids [54].

**Figure 7:**
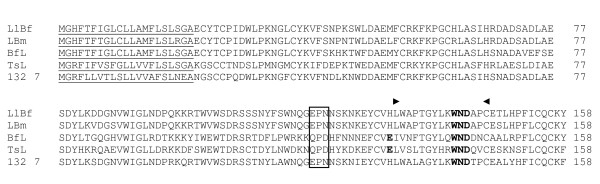
Alignment of *A. labialis *C-type lectin (clone 132) with L1Bf (*Bungarus fasciatus*), LBm (*B. multicinctus*), BFL2 (*Bungarus fasciatus*) and TsL (*Trimeressurus stejneger*) [47,53] Gln-Pro-Asp (QPD) sequence essential for galactose interaction is indicated by box and region flanked by (▶) and (◀) are residues essential for Ca^2+ ^interaction [67]. The number of clones is shown in italics and the predicted signal peptide using SignalP 3.0 is underlined.

This family has seven clones which form one cluster. The primary structure shows 71% and 74% identical to C-type lectins found in *Bungarus fasciatus *and *B. multicinctus *[53], 56% identical to galactose-binding lectin found in *Bitis arietans *[55], but less than 37% identical to C-type lectin related proteins. The deduced amino acid sequence reveals the conserved carbohydrate binding domain as well as the Ca^2+ ^interaction amino acid residues (Figure 7). The odd cysteine present is likely to involved in interchain disulphide bond [50,55]. The presence of only one cluster of 7 clones indicates that it may exist as homodimer similar to other C-type lectins. Further, phylogenetic analysis (data not shown) revealed that this putative C-type lectin is more closely related to galactose-binding proteins and forms a cluster distinct from that of C-type lectin like proteins that bind to blood coagulation factors or platelet receptors. Thus, this clone most likely encodes a homodimeric C-type lectin.

### Cellular transcripts

150 clones were identified as cellular trancripts which were grouped into 15 clusters and 1 singleton which constituted 32% of the entire library (Figure 1A). This suggests that harvesting the venom gland after 3–4 days after milking increases the chances of obtaining mostly high abundant toxin genes. We identified ribosomal proteins, creatine kinase, dehydrogenase, muscle troponin, cytochrome b, parvalbumin, heparin binding protein and protein disulphide isomerase (PDI) in this library. In addition, polyadenylated 12S and 16S rRNAs were also found. Although polyadenylation is the distinctive features of mRNA, polyadenylation of rRNA has been observed in yeast [56], Leishmania [57] as well as in other higher organisms including human [58]. These polyadenylated rRNAs are degraded subsequently through Polyadenyled-stimulated RNA degradation pathway [56,58].

### Unknown sequences

8% of the cDNA sequences (Figure 1A) of the library did not show any significant match to toxins or other metabolic genes and hence were categorized as unknown sequences. Bioinformatics analysis of these sequences showed only partial or poor homology to protein sequences of other organism. Their functional role is not known.

### Accelerated rate of deletions and insertions in toxin genes

Evolution of large multigene families in snake venom occurs through "birth and death" process [59]. Toxin genes appear to undergo duplication followed by accelerated evolution to give rise to new genes [60]. Some of the genes either get deleted from the genome due to unequal cross-over and other phenomena or become non-functional and degenerate into pseudogenes [61]. Such birth and death phenomenon allows the snake to adapt to target various preys. In *A. labialis*, we observed unusually accelerated rate of deletions and insertions in toxin cDNA clones resulting in the death of functional toxin genes. Out of the 43 cDNAs encoding toxins (Table 1), only 26 encode full length proteins. A total of 17 cDNAs encode for truncated products due to deletions (10 genes) or insertions (7 genes). Thus 39.5% of the toxin cDNAs encode non-functional products. Even the main toxin groups are severely affected. For example, six neurotoxin genes and three PLA_2 _genes were truncated. Interestingly, entire families of metalloproteinases and CRISPs appear to be lost due to deletions and insertions. Although some of the long-chain neurotoxin genes encode full length functional proteins, there are some deletions at their C-terminal ends. These observations were made not only in singletons, but also in a number of clusters of toxin genes. In contrast none of the cellular transcripts (150 clones; 32% of cloned genes) showed deletions or insertions in nucleotide level. Thus, the only toxin cDNAs show this unusually high rate of deletions and insertions in *A. labialis*. The phenomenon of such high rate of accelerated insertions and deletions in only the toxin genes (described here) as with the accelerated evolution of exons of the toxin genes [62] will be of great interest.

**Table 1:** Full length and truncated toxin genes found in the cDNA library of *A. labialis *venom gland.

Protein family	Number of genes	Full length genes	Truncated clones	
			
			Deletion	Addition
House keeping genes	16	16	0	0
Neurotoxins	19	13	4	2
Kunitz-type protease inhibitors	9	8	0	1
PLA_2_	7	4	2	1
C-type lectins	1	1	0	0
CRISPs	6	0	3	3
Metalloproteases	1	0	1	0

Recently, we showed that in marbled sea snake (*Aipysurus eydouxii*) the only neurotoxin gene has a dinucleotide deletion resulting in the loss of viable neurotoxin [17]. This explains the 50- to 100-fold decrease in venom toxicity in comparison to that of other species in the same genus. We proposed that this loss could be a secondary result of the adaptation of *Aipysurus eydouxii *to a new dietary habit – feeding exclusively on fish eggs and, thus, the snake no longer requires its venom for prey capture [61]. Further, this snake is physically smaller in size compared to *Aipysurus laevis*. Similarly, venom of *Austrelaps labialis *is relatively less toxic compared to *Austrelaps superbus *and *Austrelaps ramsayi*. The LD_50 _of *A. labialis *(1.3 mg/kg) [8] is higher as compared to its close relative *A. superbus *whose LD_50 _is 0.5 mg/kg when injected subcutaneouly [2]. Further, *A. labialis *are smaller in size compared to others. Thus, rapid rate of deletions/insertions, as with accelerated evolution of toxin genes, may have significant influence in the evolution and survival of *A. labialis*. In a previous study, Chijiwa et al. [63] showed that the protein composition of venoms from *Protobothrops flavoviridis *(formerly *Trimeresurus flavoviridis*) of Okinawa island was different compared to that of snakes from the main island. In Okinawa snakes, some of the main components, such as myotoxic PLA_2 _enzymes BPI and BPII, were absent. The genes encoding these proteins lost segments in their exon and intron and have become pseudogenes. This loss of BPI and BPII genes may not explain the overall decrease in the venom toxicity [63]. Interestingly, haemorrhagic metalloprotease, HR_1b _from the venom of Okinawa snakes is 10 fold less active. However, the reasons for this decrease in activity are not clearly understood. A detailed study of the entire transcriptome of their venom glands may help in understanding whether the observed regional variation in Okinawa snakes is similar to our findings with *A. labialis *snake. It would also be interesting to examine the transcriptome of *A. labialis *inhabiting the mainland to determine whether the observed accelerated deletions/insertions in the genes of the Kangaroo Island inhabitant is also a regional variation.

## Conclusion

In the present study we have presented the toxin profile of *A. labialis *by constructing cDNA library using its venom gland tissue. Neurotoxin and PLA_2 _are the most abundant family of toxin in this snake venom. However it is interesting to note that some of the toxin transcripts have been found to be terminated prematurely either due to insertion or deletion of nucleotides. Due to these insertion and deletion some of the genes might not code for functional proteins. Such a higher rate of insertion and deletion might be responsible for its lower toxicity and play crucial role evolution of toxin genes.

## Authors' contributions

RD and NNBT have and carried out the experiments, analyzed data and wrote the manuscript. AR has analyzed and interpreted the data. RMK is the principal investigator who designed the experiment, analyzed the data and critically reviewed the manuscript. All the authors have approved the final form of the manuscript.

## Supplementary Material

Additional file 1 Nucleotide sequences of long-chain neurotoxins showing insertions and deletions of nucleotides. Nucleotide sequences were aligned using ClustalW. Gaps are indicated with dots, insertion with arrow, deletion with asterisk and stop codon with square box. Click here for file

Additional file 2 Nucleotide sequences of PLA_2 _showing insertions and deletions of nucleotides. Nucleotide sequences were aligned using ClustalW. Gaps are indicated with dots, insertion with arrow and stop codon with square box. Click here for file

Additional file 3 Nucleotide sequences of CRISPs showing insertions and deletions of nucleotides. Nucleotide sequences were aligned using ClustalW. Gaps are indicated with dots, insertion with arrow, deletion with asterisk and stop codon with square box. Click here for file
