# Supplementary material for: Unusual accelerated rate of deletions and insertions in toxin genes in the venom glands of the pygmy copperhead (Austrelaps labialis) from kangaroo island
Source: BMC Evol Biol. 2008 Feb 28;8:70. doi: 10.1186/1471-2148-8-70 (PMC2287176; doi:10.1186/1471-2148-8-70)
Supplement: Additional file 1 — Nucleotide sequences of long-chain neurotoxins showing insertions and deletions of nucleotides. Nucleotide sequences were aligned using ClustalW. Gaps are indicated with dots, insertion with arrow, deletion with asterisk and stop codon with square box. [file 1471-2148-8-70-S1.pdf]

Additional file 1: Nucleotide sequence of long-chain neurotoxins showing insertions and deletions of nucleotides.▼, insertion; *, deletion and ▭, stop codon.

```
8A    ATGAAAACTCTGCTGCTGACCTTGGTGGTGGTGACAATCGTGTGCCTGGACTTAGGTAACAGCTTCTCATGCTACAAAACACCTCATGTTAAATCT    96
5B    ATGAAAACTCTGCTGCTGACCTTGGTGGTGGTGACAATCGTGTGCCTGGACTTAGGTAACAGCTTCTCATGCTACAAAACACCTGGTGTTAAATCT    96
160   ATGAAAACTCTGCTGCTGACCTTGGTGGTGGTGACAATCGTGTGCCTGGACTTAGGTAACAGCTTCTCATGCTACAAAACACCTCATGTTAAATCT    96
646   ATGAAAACTCTGCTGCTGACCTTGGTGGTGGTGACAATCGTGTGCCTGGACTTAGGTAACAGCTTCTCATGCTACAAAACACCTCATGTTAAATCT    96
509   ATGAAAACTCTGCTGCTGACCTTGGTGGTGGTGACAATCGTGTGCCTGGACTTAGCTGGTGGCTTGATATGCTACCTGGGACGTAATACCCCTGGG    96
21    ATGAAAACTCTGCTGCTGACCTTGGTGGTGGTGACAATCGTGTGCCTGGACTTTGGTGGTGGCTTGATATGCTACATGGGACCTAAAACCCCTCGG    96
242   ATGAAAACTCTGCTGCTGACCTTGGTGGTGGTGACAATCGTGTGCCTGGACTTAGGTGACAGCTTGAGATGCTACATGGGACCTAAAACCCCTCGG    96
112   ATGAAAACTCTGCTGCTGACCTTGGGGGTGGTGACAATCGTATGCCTGGACTTAGGTAACAGCTTCTCATGCTACAAAACACCTGATGTTAAATCT    96

                     *                  **     **
8A    GAGCCTTGTGCACCTGGGCAGAACCTATGCTATACAAAGACTTGGTGTGATCGTTTTTGTTCCATCAGAGGAAAGGTAATCGAACTGGGATGTGCT    192
5B    GAGCCTTGTGCACCTGGGGAGAACCTATGCTATACAAAGACTTGGTGTGATGCTTTTTGTGGCGACAGAGGAAAGCGAGTTGAAATGGGATGTGCT    192
160   GAGCCTTGTGCACCTGGGCAGAACCTATGCTATAC.AAGACTTGGTGTGATCGTTTTTGTTCCATCAGAGGAAAGG[TAA]TCGAACTGGGATGTGCT    191
646   GAGCCTTGTGCACCT.GGCAGAACCTATGCTATACAAAGACTTGGTGTGATCGTTTTTGTTCCATCAGAGGAAAGG[TAA]TCGAACTGGGATGTGCT    191
509   A...CTTGTCCACCTGGGCAGGAGCTATGCTACATA.GTGG..GGTGTGGTGCTTTTTGTCACGTCAGAAG......AGTCAAATTGGGATGTGCT    180
21    A...CTTGTCCACCTGGGCAGAACCTATGCTATACAAAGACTTGGTGTGATGGTTTTTGTGGCAGCAGAGGAAAGGTAGTCGTATTGGGATGTGCT    189
242   A...CTTGTCCACCTGGGGAGAACCTATGCTTTACAAAGACTTGGTGTGATCCTCGGTGTAGCCTCCTAGGAAAGTTAGTCAAATTGGGATGTGCT    189
112   GAGCCTTGTGCACCTGGGGAGAACCTATGCTATACAAAGACTTGGGGTGATCGTTTTTGTTCCATCAGAGGAAAGGTAATCGAACTGGGATGTGCT    192

                         *                                               ** ▼▼        *▼▼
8A    GCTACTTGCCCTCCAGCGGAGCCAAAAAAGGATATTACCTGTTGCTCAACAGACAATTGCAACCCACATCCG[TAA]...................    267
5B    GCTACTTGCCCTACAGTGAAGCCCGGTGTGGATATTACCTGTTGCTCAACAGACAATTGCAACCCATTTCC..AA..CACACAACTCTCGT[TGA]..    282
160   GCTACTTGCCCTCCAGCGGAGCCAAAAAAGGATATTACCTGTTGCTCAACAGACAATTGCAACCCACATCCGTAA..CAGAAAA..CTCGTTGA..    281
646   GCTACTTGCCCTCCAGCGGAGCCAAAAAAGGATATTACCTGTTGCTCAACAGACAATTGCAACCCACATCCGTAA..CAGAAAA..CTCGTTGA..    281
509   GCTACTTGCCCTATACCGAAGCGGTATGAGAAGGTTACCTGTTGCTCAACAGACAATTGCAACCAATTTCCG..ACACAGAAA..TCTCGT[TGA]..    270
21    GCTACTTGCCCTACAGTGAAGCCCGGTGTGGATATTACCTGTTGCGCAACCGACAAGTGCAACCCATTTCCAAAA..ACGAAAG..CTCCTTGGGA    281
242   GCTACTTGCCCTATACCGAAGTCCTATGAGGATGTTACCTGTTGCTCAACAGACAACTGCAACAGATTTCCGAAATGGGAACGTTCTAGACCACGT    285
112   GCTACTTGCCCTCCAGCGGAGCC.AAAAAGGATATTACCTGTTGCTCAACAGACAATTGCAACCCACATCCGTAACAGAAAAAAAGTTGAGTTTTG    287

8A    ................................................................................................    267
5B    ................................................................................................    282
160   ................................................................................................    281
646   ................................................................................................    281
509   ................................................................................................    270
21    ACGTCCT[TGA]..........................................................................................    291
242   CCTCGAGGGTTGCTCTCATCCATCATGGACCATCCT[TGA]...........................................................    324
112   CTCTCATCCATCATGGACCATCCTTGAAAATTTATGCTTGTGGCCTTTACCGCCAGATGGTCCATCATCCCCCTCTCCCCTGCTGTCTTTGACATC    383

8A    ................................................................................................    267
5B    ................................................................................................    282
160   ................................................................................................    281
646   ................................................................................................    281
509   ................................................................................................    270
21    ................................................................................................    291
242   ................................................................................................    324
112   TCAACATCTTTCCCTTTTCTCTCGTTCTGTACGTTTCCTTCTGCTAGTTCTG[TAG]..........................................    438
```
