# Supplementary material for: Unusual accelerated rate of deletions and insertions in toxin genes in the venom glands of the pygmy copperhead (Austrelaps labialis) from kangaroo island
Source: BMC Evol Biol. 2008 Feb 28;8:70. doi: 10.1186/1471-2148-8-70 (PMC2287176; doi:10.1186/1471-2148-8-70)
Supplement: Additional file 2 — Nucleotide sequences of PLA2 showing insertions and deletions of nucleotides. Nucleotide sequences were aligned using ClustalW. Gaps are indicated with dots, insertion with arrow and stop codon with square box. [file 1471-2148-8-70-S2.pdf]

Additional file 2: Nucleotide sequence of PLA_2 showing insertion.
▼, insertion and ▭, stop codon.

```
636   ATGTATGCTGCTCACCTTCTGGTCCTGTTGGTAGTTTGTGTCTCCCTCTTAGGAGCTGCCAGCATTCCTCCGCAGCCTCTCAACCTCGTACAATTC    96
518   ATGTATCCTGCTCACCTTCTGGTCCTGTTGGCAGTTTGTGTCTCCCTCTTAGGAGCTGCCAGCATTCCTCCGCAGCCTCTCAACCTCGTACAATTC    96

636   AGCTACTTGATTCAATGTGCCAACCATGGCAGTCGAGCTACTTGGCATTATACGGACTACGGTTGCTACTGCGGCTCAGGAGGTAGCGGGACACCC   192
518   GGCTACTTGATTCAATGTGCCAACCATGGCAGTCGAGCTACTTGGCATTATACGGACTACGGTTGCTACTGCGGCTCAGGAGGTAGCGGGACGCCC   192

                                                                    ▼
636   GTGGATGAGTTGGATAGGTGCTGCCAGACTCATGACAACTGCTATGCTGAAGCCG.AAAAAAAAGGATGCTACCCCAAGATATCGGCGTATGATTA   287
518   GTGGATGAGTTGGATAGGTGCTGCCAGACTCATGACAACTGCTATGGTGAAGCCGAAAAAAAAAGGATGCTACCCCAAGATGTCGGCGTA[TGA]TTA   288

636   CTACTGTGGCGAAAATGGACCCTACTGCAGAAATATCAAAAAGGAGTGTCAACGTTTTGTGTGTGATTGTGACGTCGAAGCAGCCTTCTGCTTTGC   383
518   CTACTGTGGCGAAAATGGACCCTACTGCAGAAATATCAAAAAGGAGTGTCAACGTTTTGTGTGTGATTGTGACGTCGAAGCAGCCAAGTGCTTTGC   384

636   CAGAGCCCCTTACAACGACGCGAACTGGAATATCGACACCAAGACACGTTGTCAA[TGA]   441
518   CAGAGCCCCTTACAACGACGCGAACTGGAATATCGACACCAAGAAACGTTGTCAATGA   442
```
