# Supplementary material for: Unusual accelerated rate of deletions and insertions in toxin genes in the venom glands of the pygmy copperhead (Austrelaps labialis) from kangaroo island
Source: BMC Evol Biol. 2008 Feb 28;8:70. doi: 10.1186/1471-2148-8-70 (PMC2287176; doi:10.1186/1471-2148-8-70)
Supplement: Additional file 3 — Nucleotide sequences of CRISPs showing insertions and deletions of nucleotides. Nucleotide sequences were aligned using ClustalW. Gaps are indicated with dots, insertion with arrow, deletion with asterisk and stop codon with square box. [file 1471-2148-8-70-S3.pdf]

Additional file 3: Nucleotide sequence of CRISPs showing insertions and deletions of nucleotides.▼, insertion; *, deletion and ▭, stop codon.

```
Oxyuranus   ATGATTGCCTTCATTGTCTTGCTAAGTCTTGCTGCAGTGCTGCAACAGTCTTCTGGAACTGTTGATTTTGCTTCTGAGTCAAGTAACAAAAAAGAT   96
521         ATGATTGCCTTCATTGTCTTGCTAAGTCTTGCTGCAGTGCTGCAACAGTCTTCTGGAACTGTTGATTTTGCTTCTGAGTCAAGTAACAAAAAAGAT   96
218         ATGATTGCCTTCATTGTCTTGCTAAGTCTTGCTGCAGTGCTGCAACAGTCTTCTGGAACTGTTGATTTTGCTTCTGAGTCAAGTAACAAAAAAGAT   96
492         ATGATTGCCTTCATTGTCTTGCTAAGTCTTGCTGCAGTGCTGCAACAGTTTTCTGGAACTGTTGATTTTGCTTCTGAGTCAAGTAACAAAAAAGAT   96
399         ATGATTGCCTTCATTGTCTTGCTAAGTCTTGCTGCAGTGCTGCAACAGTCTTCTGGAACTGTTGATTTTGCTTCTGAGTCAAGTAACAAAAAAGAT   96
217         ATGATTGCCTTCATTGTCTTGCTAAGTCTTGCTGCAGTGCTGCAACAGTCTTCTGGAACTGTTGATTTTGCTTCTGAGTCAAGTAACAAAAAAGAT   96
363         ATGATTGCCTTCATTGTCTTGCTAAGTCTTGCTGCAGTGCTGCAACAGTCTTCTGGAACTGTTGATTTTGCTTCTGAGTCAAGTAACAAAAAAGAT   96

Oxyuranus   TACCGAAAGGAGATTGTTGACAAGCACAATGATTTAAGGAGATCAGTGAAACCAACTGCTAGGAACATGTTACAAATGAAATGGAATTCTCGTGCT   192
521         TACCGAAAGGAGATTGTTGACAAGCACAATGCTTTAAGGAGATCAGTGAAACCAACTGCTAGGAACATGTTACAAATGGAATGGAATTCTCGTGCT   192
218         TACCGAAAGGAGATTGTTGACAAGCACAATGCTTTAAGGAGATCAGTGAAACCAACTGCTAGGAACATGTTACAAATGGAATGGAATTCTCGTGCT   192
492         TACCGAAAGGAGATTGTTGACAAGCACAATGCTTTAAGGAGATCAGTGAAACCAACTGCTAGGAATATGTTACAAATGGAATGGAATTCTCGTGCT   192
399         TACCGAAAGGAGATTGTTGACAAGCACAATGCTTTAAGGAGATCAGTGAAACCAACTGCTAGGAACATGTTACAAATGGAATGGAATTCTCGTGCT   192
217         TACCGAAAGGAGATTGTTGACAAGCACAATGCTTTAAGGAGATCAGTGAAACCAACTGCTAGGAACATGTTACAAATGGAATGGAATTCTCGTGCT   192
363         TACCGAAAGGAGATTGTTGACAAGCACAATGCTTTAAGGAGATCAGTGAAACCAACTGCTAGGAACATGTTACAAATGGAATGGAATTCTCGTGCT   192

Oxyuranus   GCTCAAAATGCAAAACGTTGGGCAAATAGATGTACTTTTGCTCACAGTCCACCATACACAAGAACTGTGGGAAAACTCCGTTGTGGTGAAAATATA   288
521         GCTCAAAATGCAAAACGTTGGGCAGATAGATGTACTTTTGCTCACAGTCCACCACATACAAGAACTGTGGGAAAACTCCGTTGTGGTGAAAATATA   288
218         GCTCAAAATGCAAAACGTTGGGCAGATAGATGTACTTTTGCTCACAGTCCACCACATACAAGAACTGTGGGAAAACTCCGTTGTGGTGAAAATATA   288
492         GCTCAAAATGCAAAACGTTGGGCAGATAGGTGTACTTTTGCTCACAGTCCACCACATACAAGAACTGTGGGAAAACTCCGTTGTGGTGAAAATATA   288
399         GCTCAAAATGCAAAACGTTGGGCAGATAGATGTACTTTTGCTCACAGTCCACCACATACAAGAACTGTGGGAAAACTCCGTTGTGGTGAAAATATA   288
217         GCTCAAAATGCAAAACGTTGGGCAGATAGATGTACTTTTGCTCACAGTCCACCACATACAAGAACTGTGGGAAAACTCCGTTGTGGTGAAAATATA   288
363         GCTCAAAATGCAAAACGTTGGGCAGATAGATGTACTTTTGCTCACAGTCCACCACATACAAGAACTGTGGGAAAACTCCGTTGTGGTGAAAATATA   288

                                                                 ▼       ▼
Oxyuranus   TTCATGTCAAGTCAACCTTTTGCATGGAGTGGAGTAGTTCAGGCTTGGTATGA.TGAAGTC.AAAAAATTTGTCTATGGCATTGGAGCAAAGCCAC   382
521         TTCATGTCAACTCAACCTTTTGCATGGAGTGGCGTAGTTCAGGCTTGGTATGA.TGAAGTC.AAAAAATTCGTCTATGGCATTGGAGCAAAGCCAC   382
218         TTCATGTCAACTCAACCTTTTGCATGGAGTGGCGTAGTTCGGGCTTGGTATGA.TGAAGTC.AAAAAATTCGTCTATGGCATTGGAGCAAAGCCAC   382
492         TTCATGTCAACTCAACCTTTTGCATGGAGTGGCGTAGTTCAGGCTTGGTATGA.TGAAGTC.AAAAAATTCGTCTATGGCATTGGAGCAAAGCCAC   382
399         TTCATGCCAACTCAACCTTTTGCATGGAGTGGCGTAGTTCAGGCTTGGTATGA.TGAAGTCAAAAAAATTCGTCTATGGCATTGGAGCAAAGCCAC   383
217         TTCATGCCAACTCAACCTTTTGCATGGAGTGGCGTAGTTCAGGCTTGGTATGA.TGAAGTCAAAAAAATTCGTCTATGGCATTGGAGCAAAGCCAC   383
363         TTCATGCCAACTCAACCTTTTGCATGGAGTGGCGTAGTTCAGGCTTGGTATGATTGAAGTCAAAAAAATTCGTCTATGGCATTGGAGCAAAGCCAC   384

Oxyuranus   CAAGTTCTGTTATTGGCCATTATACCCAGGTAGTTTGGTACAAAAGTCACCTTCTTGGTTGTGCTTCTGCCAAATGTTCTTCAACCAAATACCTCT   478
521         CAGGTTCTGTTATTGGCCATTATACCCAGGTAGTTTGGTACAAAAGTCACCTTCTTGGTTGTGCTTCTGCCAAATGTTCTTCAACCAAATACCTCT   478
218         CAGGTTCTGTTATTGGCCATTATACCCAGGTAGTTTGGTACAAAAGTCACCTTCTTGGTTGTGCTTCTGCCAAATGTTCTTCAACCAAATACCTCT   478
492         CAGGTTCTGTTATTGGCCATTATACCCAGGTAGTTTGGTACAAAAGTCACCTTCTTGGTTGTGCTTCTGCCAAATGTTCTTCACCCAAATACCTCT   478
399         CAGGTTCTGTTATTGGCCATTATACCCAGGTAGTTTGGTACAAAAGTCACCTTCTTGGTTGTGCTTCTGCCAAATGTTCTTCAACCAAATACCTCT   479
217         CAGGTTCTGTTATTGGCCATTATACCCAGGTAGTTTGGTACAAAAGTCACCTTCTTGGTTGTGCTTCTGCCAAATGTTCTTCAACCAAATACCTCT   479
363         CAGGTTCTGTTATTGGCCATTATACCCAGGTAGTTTGGTACAAAAGTCACCTTCTTGGTTGTGCTTCTGCCAAATGTTCTTCAACCAAATACCTCT   480

               ▼                                                     *            **                    *  *
Oxyuranus   ACG.TTTGTCAATACTGCCCAGCAGGGAACATCATAGGTTCAATTGCTACTCCATATAAATCAGGACCACCTTGTGGGGACTGTCCTTCGGCTTGT   573
521         ACG.TTTGTCAATACTGCCCAGCAGGGAACATCAGAGGTTCAATTGCTACTCCATAT.AATCAGGCCCGC..TGGTGGGACTGTCCTTCGGCTGG.   569
218         ACG.TTTGTCAATACTGCCCAGCAGGGAACATCAGAGGTTCAATTGCTACTCCATATAAATCAGGGCCAGCTTGTGGGGACTGTCCTTCGGCTTGT   573
492         ACG.TTTGTCAATACTGCCCAGCAGGGAACATCAGAGGTTCAATTGCTACTCCATATAAATCAGGGCCAGCTTGGGGGGACTGTCCTTCCGCTTGT   573
399         ACCTTTTGTCAATACTGCCCAGCAGGGAACATCAGAGGTTCAATTGCTACTCCATATAAATCAGGGCCAGCTTGTGGGGACTGTCCTTCGGCTTGT   575
217         ACG.TTTGTCAATACTGCCCAGCAGGGAACATCAGAGGTTCAATTGCTACTCCATATAAATCAGGGCCAGCTTGTGGGGACTGTCCTTCGGCTTGT   574
363         ACG.TTTGTCAATACTGCCCAGCAGGGAACATCAGAGGTTCAATTGCTACTCCATATAAATCAGGGCCAGCTGGTGGGGACTGTCCTTCGGC.TGT   574

               *               * *        *   *          * *                        *
Oxyuranus   GACAACGGACTATGCACAAATCCTTGCAAACATAACGATGACTTATCGAACTGCAAACCTTTAGCGAAAAAAAGTAAATGCCAGACTGAATGGATC   669
521         GTC.ACGGACTATGCCCAA.T.CTTGCAAA.ATGAAGATGCCTTCCC.AACTGCAAGGCTTTACCGAAAAAAACTAAATGCAAGACTGAATGGATC   660
218         GTC.ACGGACTATGCACAAATCCTTGCAAATATGAAGATGCCTTCACGAACTGCAAAGCTTTGGCGAAAAAA.CTAAATGCAAGACTGAATGGATC   667
492         GTC.ACGGACTATGCACAAATCCTTGCAGATATGAAGATGCCTTCCC.AACTGCAAGGCTTTACCGAAAAAAACTAAATGCAAGACTGAATGGATC   667
399         GTCACCGGACTATGCACAATCCTTGCAAAATATGAAGATGCCTTCACGAACTGCAAAGCTTTAGCGAAAAAAACTAAATGCAAGACTGAATGGATC   671
217         GTCAACGGACTATGCACAAATCCTTGCAAATATGAAGATGCCTTCACGAACTGCAAAGCTTTAGCGAAAAAAACTAAATGCAAGACTGAATGGATC   670
363         GTC.ACGGACTATGCCCAAATCCTTGC.AATATG.AGATGCCTTC.CGAACTGCAAAGCTTTAGCGAAAAAAACTAAATGCAAGACTGAATGGATC   666

Oxyuranus   AAGTCAAAATGTCCTGCTACTTGCTTCTGCCGCACTGAAATAATATAG.....   717
521         AAGTCAAAATGCCCTGCTACTTGCTTCTGCCACAATAAAATAATATAG.....   708
218         AAGTCAAAATGCCCTGCTACTTGCTTCTGCCACAATAAAATAATATAG.....   715
492         AAGTCAAAATGCCCTGCTACTTGCTTCTGCCACAATAAAATAATATAG.....   715
399         AAGTCAAAATGCCCTGCTACTTGCTTCTGCCACAATAAAATAATATAG.....   719
217         AAGTCAAAATGCCCTGCTACTTGCTTCTGCCACAATAAAATAATATAG.....   718
363         AAGTCAAAATGCCCTGCTACTTGCTTCTGCCACAATAAAATAATATAG.....   714
```
